# Occupational COVID-19: what can be learned from notifications of occupational diseases?

**DOI:** 10.1136/oemed-2020-107121

**Published:** 2020-11-06

**Authors:** Henk F van der Molen, Sanja Kezic, Steven Visser, Gerda de Groene, Jaap Maas, Astrid de Wind, Sietske Tamminga

**Affiliations:** Amsterdam UMC, University of Amsterdam, Public and Occupational Health, Netherlands Center for Occupational Diseases, Amsterdam Public Health Research Institute, Amsterdam, The Netherlands

**Keywords:** occupational health

Marinaccio and colleagues are applauded for providing valuable data on the risk of acquiring COVID-19 due to exposure in the workplace in Italy.^1^ Once again, this emphasises the importance of an assessment of the working circumstances and registration of occupational diseases. Data on work-related COVID-19 in the study of Marinaccio and colleagues was based on compensation claims of workers. In the Netherlands, an expert-based one-page guidance document for assessment and reporting of COVID-19 as occupational disease was developed by occupational physicians and data specialists. This guidance document, based on the general 6-step approach,^2^ was published on the website of the Netherlands Center for Occupational Diseases^3^ and communicated by newsletters. Occupational COVID-19 reports, International Classification of Diseases-10 code U.071, contain information about age, sex, occupation, economic sector, work disability and potential cause for occupational COVID-19. In the period March–September 2020, 904 cases were reported.

Although this number is much lower compared to claims received in Italy reported by the Italian Workers’ Compensation Authority, in the Netherlands, COVID-19 was the 2nd most common occupational disease reported from March-September (33% of all reported illnesses), exceed only by mental illness (42%). Musculoskeletal disorders accounted for 19%. This percentage is consistent with their estimate of 30% of reported occupational COVID-19 cases in the working age. Most Dutch occupational COVID-19 cases were reported within the nurse and caring homes (713; 79%) and hospitals (125; 14%), which is in line with the 70% reported for healthcare workers in Ontario Canada.^4^ The other cases were mainly from the sectors ‘wholesale and retail trade’, ‘administrative and support service activities’, ‘manufacturing’, ‘public administration and defence’ and ‘education’. Work disability was reported in most of the cases and in 21% of the workers this lasted for more than 3 months. Among workers from nurse and caring homes, contact with infected patients and colleagues was reported in 94% of the cases. In 516 of the 670 cases (77%), unprotected contact with a patient with COVID-19 was reported, which could be caused by a lack of sufficient personal protective equipment. In 27 of the 670 cases (4%), nurses and caregivers were infected while using personal protective equipment.

Both approaches in assessment and reporting of occupational COVID-19 cases have own (dis)advantages. Compensation claim data may be biassed by the financial need of a worker^1 4^ while the registry of occupational diseases may be biassed by the reporting behaviour of occupational physicians.^5^ Nevertheless, notification and accompanying data are important to explore the impact of work-related risk factors and to identify groups at high risk of contracting an occupational disease. This is particularly relevant for new emerging occupational diseases such as COVID-19, when the knowledge on risk factors is limited but implementation of prevention measures is urged.

Recording of occupational diseases is of additional value in developing preventive measures in the workplace. To enable comparison of notification approaches used in different countries, harmonisation of epidemiological surveillance systems and case definitions such as currently done by the OMEGA-NET initiative^6^ is of the utmost importance.
